# Knowledge, attitudes, and practices regarding contraception amongst community pharmacy staff: a cross-sectional study in Nigeria

**DOI:** 10.3389/frph.2025.1488707

**Published:** 2025-03-24

**Authors:** Obi Peter Adigwe, Godspower Onavbavba

**Affiliations:** National Institute for Pharmaceutical Research and Development, Federal Capital Territory, Abuja, Nigeria

**Keywords:** pregnancy, abortion, contraceptives, birth control, family planning

## Abstract

**Introduction:**

Lack of access to effective contraception methods can lead to an increased prevalence of unintended pregnancies, as well as possible deleterious health consequences. Community pharmacies represent the most accessible gateway for population medicines' and healthcare needs. Regarding contraceptives, they have also been identified as a platform for the provision of additional services, such as complementary counselling. This study aimed at assessing the knowledge, attitudes, and practices of community pharmacy staff towards contraception.

**Methods:**

A cross-sectional study was undertaken in the Federal Capital Territory, Nigeria. Data were collected from 315 community pharmacy staff using self-administered questionnaires. The participants' knowledge and attitude scores were categorised using Bloom's cut-off point. Analyses were undertaken using Statistical Package for Social Sciences. The data were analysed using frequency distribution, chi-square, and linear regression at a 5% level of significance.

**Results:**

Male participants in the study (165/315, 52.4%) were slightly higher than the female respondents (150/315, 47.6%), and about two-thirds of the study cohort were pharmacists (200/315, 63.5%). The majority of the participants (183/315, 58.1%) had poor knowledge of contraceptive use. A quarter of them (81/315, 25.7%) reported moderate attitudes. Almost all the participants (279/298, 93.6%) indicated recommending contraceptives for married adults, and a significant proportion of them (136/292, 45.5%) were opposed to recommending contraceptives for unmarried adolescents. Study respondents' professional role was also identified as a significant influence on their knowledge of contraceptives and contraception (*p* < 0.001).

**Conclusion:**

Findings from this study revealed poor knowledge and negative attitudes of community pharmacy staff towards contraception. Government and relevant stakeholders can build on these novel findings to reform pertinent contextual policies and practices. This can significantly improve access to contraceptives amongst the populace, and consequently reduce unintended pregnancies alongside possible health and societal implications.

## Introduction

Globally, an estimated 250 million pregnancies occur each year, with up to one-third of these pregnancies being unintended (1, 2). Unintended refers to unwanted or mistimed pregnancies which can occur due to failure to choose an effective contraception method, or incorrect use of contraceptives (3, 4). Such pregnancies can place an economic burden on societies, as well as lead to reproductive health risks for women (5). Close to a quarter of unwanted pregnancies are terminated using unsafe methods, and up to 18% end up in unplanned births (6). Whilst unsafe termination of pregnancies is common in low-income countries, available evidence suggests that the abortion rates in developing and developed countries are of a similar proportion (7). In Nigeria, between 2015 and 2019, 10.5 million pregnancies occur annually, with 2.99 million being unintended, and 1.43 million ending in abortion (7). These high abortion rates alongside associated safety concerns in low-income countries highlight the need for improved access to contraception, especially from frontline healthcare practitioners in community pharmacy practice.

Contraception, also known as birth control, anticonception, and fertility control, refers to various relevant methods used for the prevention of pregnancies. Contraception has been in use for a long time; however, effective and safe methods of birth control only became available in the 20th century (8). Methods such as sterilisation, the use of intrauterine devices, and implantable birth control have been identified as the most effective means of fertility control, and this list is followed by other methods such as oral pills, patches, vaginal rings, and injections (9). Other techniques that are categorised as less effective include barrier methods, such as the use of condoms, diaphragm, and birth control sponges. The least effective birth control methods are the use of spermicides and withdrawal methods. Pregnancies in teenagers are associated with a greater risk of poor outcomes (10). Promoting comprehensive sex education and access to contraceptives for this age group decreases the rate of unwanted pregnancies (11). Birth control can improve women's delivery outcomes and the survival of their children by increasing the length of time between pregnancies (12).

In Nigeria, contraceptive use is shaped by a complex interplay of cultural and systemic factors (13). Deeply rooted religious beliefs and societal stigma often create significant barriers, discouraging open discussions about contraception and complicating access for individuals seeking options (14). These challenges are further exacerbated by gender dynamics, where family planning decisions are frequently dominated by men, limiting women's autonomy in making informed choices (15).

The public can experience barriers in accessing contraceptives, especially when some health providers feel that certain individuals that are unmarried should not receive such services due to their personal belief. These barriers can manifest in various forms, including biased attitudes, lack of comprehensive training on reproductive health, and inadequate communication about contraceptive options (16). Providers may perpetuate stigma by questioning the moral or social appropriateness of contraceptive use among certain populations, leading to discriminatory practices that deny essential services (17). Additionally, the insufficient integration of family planning education into healthcare training programs means that many practitioners lack the knowledge necessary to provide unbiased, evidence-based information about contraceptives (18). As a result, these barriers not only limit access to contraceptives but also undermine broader public health efforts aimed at reducing unintended pregnancies and promoting reproductive rights in Nigeria.

The community pharmacy setting represents an essential point for accessing contraceptives. This setting also serves as a venue for offering counselling services to the public as well as a first point of call for issues relating to birth control pills (19–21). Community pharmacies play a pivotal role in access to contraceptives, and this can be influenced by the knowledge and attitudes of the healthcare personnel practicing in this setting. Appropriate knowledge, positive attitudes, and good practices are critical elements that can enable a community pharmacy staff to provide comprehensive counselling, as well as create necessary awareness regarding contraceptives, thus preventing unintended pregnancies (22, 23). Several studies have been undertaken in relation to knowledge, attitudes, and practices towards contraceptive use in Nigeria (24, 25), there is however paucity of information about the community pharmacy staff in this area. It is against this backdrop that this study aimed at assessing the knowledge, attitudes, and practices of community pharmacy staff towards contraception.

## Methods

### Study design

The study was undertaken between May and August 2022 in Nigeria's Federal Capital Territory using a cross-sectional study design. The data collection tool (Supplementary File) was developed following an extensive literature review (5, 26–29). The items in the instrument were knowledge, attitudes, and practices towards contraceptives, as well as a section on socio-demographic characteristics. The study tool was structured to assess community pharmacy staff in these thematic areas. The items assessing knowledge were answered on a “true/false” basis and an additional “I do not know” option. The questions assessing attitudes were structured as “agree”, “disagree”, and “not sure”, whilst practice questions were answered on a “yes” or “no” basis.

### Validation of research instrument

Questionnaire validation was undertaken by an expert panel comprising faculty members engaged in research activities in the field of contraception. These experts were chosen from various institutions, and were made of individuals who are familiar with survey design, validation processes, research methodologies as well as psychometrics and construct validity. Face and content validations were undertaken. The study tool was assessed for appropriateness, complexity, attractiveness, and relevance of the items. Content validity ratio and content validity index tests were undertaken for each item, and only those that passed these tests were included in the questionnaire. Cronbach alpha's test was also conducted to assess the reliability of the questionnaire, and this gave a value of 0.83, indicating internal consistency in the questionnaire items. The questionnaire was pilot-tested by administering it to an initial cohort of 20 participants who were randomly selected. The feedback received did not necessitate any further change, and this led to the final version of the questionnaire.

### Sampling

According to a study by Ekpenyong et al*.* (30), there are 455 registered community pharmacies in the Federal Capital Territory. A minimum sample size of 314 was calculated for an estimated number of 1,700 pharmacists and pharmacy support staff in the Federal Capital Territory. This was computed at 95% confidence level, 5% margin of error, and 50% response distribution using Epi Info software version 7 (31). This is in tandem with Cochran's formula for calculating the sample size of a finite population (32). The sample size was rounded up to 400 to account for non-response. Participants were recruited following a convenience sampling strategy deployed across community pharmacies in the Federal Capital Territory to get enough respondents. Inclusion criteria adopted for the study include pharmacists licensed to practice; trained pharmacy technicians; and support staff involved in roles pertaining to dispensing. Pharmacy staff who did not have any role to play in relation to dispensing medications to patients were excluded from the study. Paper-based questionnaires were administered to the study participants.

### Ethics consideration

Prior to the data collection phase, ethical approval was obtained from the Federal Capital Territory Health Research Ethics Committee (Approval number: FHREC/2021/01/97/12-08-21), and participation in the study was voluntary. Written informed consent was obtained from the participants before administering the questionnaires. Confidentiality and anonymity were strictly maintained throughout the data collection process. All information that could link participants to their responses was not included in the data collection tool.

### Data analysis

Following the retrieval of questionnaires, data were entered into Statistical Package for Social Sciences version 25. Descriptive statistical analyses were undertaken. For the knowledge section, each correct response was assigned a score of 1, and incorrect responses and unanswered questions were assigned 0. The participants' overall knowledge score was categorised using Bloom's cut-off point as good for 80%–100%, moderate for 60%–79%, and poor for ≤59% (33). Bloom's cut-off point was chosen because it provides a widely accepted framework for interpreting survey scores, ensuring consistency and comparability across studies on knowledge assessment. For the attitude section, questions were assigned 1 point for a positive attitude towards contraception, and 0 was assigned for unanswered or negative feedback. The total attitude score for each participant was categorised by also using Bloom's cut-off as positive for 80%–100%, moderate for 60%–79%, and negative for ≤59%. Findings from the practice section were presented as percentages and frequencies as this approach is more appropriate for capturing behavioral trends.

Student's *t*-test and analysis of variance (ANOVA) were undertaken to determine relationships between mean knowledge scores and socio-demographic characteristics. *post hoc* analysis (LSD) was performed in cases of significant ANOVA tests for multiple comparisons. A *p*-value of 0.05 or less represented the threshold for statistical significance. Linear regression was used with 95% confidence interval to show the strengths of association. Finally, a *p-*value of less than 0.05 in the multivariate regression analysis was used to identify variables significantly associated with the knowledge of contraception amongst community pharmacy staff.

## Results

### Demography

A total of 315 community pharmacy staff comprising (165/315, 52.4%) male participants and (150/315, 47.6%) female respondents participated in the study. Close to half of the sample (149/315, 47.3%) were between the ages of 30–39 years. Slightly above a third of the participants (114/315, 36.2%) were educated up to postgraduate level. Further details on the socio-demographic characteristics of the respondents are provided in Table 1.

**Table 1:** Socio-demographic characteristics.

Variable	Frequency (%)
Gender	
Male	165 (52.4)
Female	150 (47.6)
Age	
<20	6 (1.9)
20–29	100 (31.7)
30–39	149 (47.3)
40–49	26 (8.3)
50 and above	34 (10.8)
Highest level of education	
Secondary education	7 (2.2)
National diploma/NCE	31 (9.8)
First degree	163 (51.7)
Postgraduate	114 (36.2)
Position	
Pharmacist	200 (63.5)
Pharmacy technician	16 (5.1)
Nurse	43 (13.7)
CHEW	29 (9.2)
Pharmacy support staff	27 (8.6)
Years of experience	
<5	102 (32.4)
5–10	130 (41.3)
>10	83 (26.3)

### Knowledge

The total mean knowledge score for the participants was 11.55 ± 3.33 (range; 1–21). The majority of the participants (183/315, 58.1%) had poor knowledge regarding contraception, a third of the participants (110/315, 34.9%) reported moderate knowledge, whilst only a few of the respondents (22/315, 7.0%) had good knowledge.

In terms of medical eligibility criteria for contraceptive use, a strong majority of the participants (273/315, 86.7%) indicated correctly that the eligibility criteria provide guidance regarding which clients can use contraception methods safely. About three-quarters (231/315, 73.3%) of the participants knew that cigarette smoking could increase the risk of serious cardiovascular problems from combined oral contraceptive use. Only a third of the participants were fully knowledgeable about hormonal contraceptives' lack of association (108/315, 34.3%) with permanent infertility. Further details on knowledge of contraceptives are provided in Table 2.

**Table 2:** Knowledge regarding use of contraceptives.

SN	Statement	True	False	I don't know
1	Medical eligibility criteria for contraceptive use provides guidance regarding persons that can use contraceptive methods safely	273 (87.5)	26 (8.3)	13 (4.2)
2	Medical eligibility criteria for contraceptive use is only considered when the contraceptive to be administered is parenteral.	106 (34.4)	180 (58.4)	22 (7.1)
3	There could be delay in returning to full fertility after discontinuation of parenteral hormonal contraceptive.	216 (70.6)	71 (23.2)	19 (6.2)
4	In some cases, permanent infertility can occur as a result of hormonal contraceptive use.	151 (50.0)	108 (35.8)	43 (14.2)
5	Cigarette smoking can increase the risk of serious cardiovascular problems from combined oral contraceptive use.	231 (75.2)	39 (12.7)	37 (12.1)
6	Progestogens-only pills are less effective than combined pills.	157 (51.6)	102 (33.6)	45 (14.8)
7	Progestogens-only pills may be recommended when oestrogen is contraindicated.	196 (65.1)	53 (17.6)	52 (17.3)
8	Long term use of combined oral contraceptives is associated with reduced risk of endometrial and ovarian cancer.	146 (48.8)	100 (33.4)	53 (17.7)
9	Oral contraceptives belong to OTC medication.	181 (60.7)	93 (31.2)	24 (8.1)
10	Combined oral contraceptive is most effective if started at day 1 of menstrual cycle.	180 (61.6)	67 (22.9)	45 (15.4)
11	Emergency oral contraceptive when taken immediately after unprotected sexual intercourse has the same level of effectiveness when taken 72 h after unprotected sexual intercourse.	127 (41.1)	159 (51.5)	23 (7.4)
12	Male condom should be removed while the penis is still erect	160 (53.3)	92 (30.7)	48 (16.0)
13	Spermicide is applied on the surface of the penis prior to intercourse	146 (49.0)	96 (32.2)	56 (18.8)
14	Compared to methods like IUDs, male condoms, and hormonal contraceptives, coitus interruptus method is more effective in preventing pregnancy	131 (43.1)	145 (47.7)	28 (9.2)
15	Emergency oral contraceptive works by preventing ovulation from occurring	131 (44.3)	140 (47.3)	25 (8.4)
16	Emergency oral contraceptive contain higher doses of hormone as compared to regular pills	167 (56.0)	84 (28.2)	46 (15.4)
17	Sterilization is a method of contraception that is easily reversible for both male and female	109 (36.3)	162 (54.0)	29 (9.7)
18	Contraception is an effective means for family planning	231 (75.7)	59 (19.3)	15 (4.9)
19	Vasectomy is the most effective permanent form of contraception available to men.	193 (62.9)	71 (23.1)	43 (14.0)
20	The procedure for vasectomy is minimally invasive	131 (43.4)	79 (26.2)	92 (30.5)
21	Tubal litigation is a female sterilization in which the fallopian tubes are permanently blocked or removed	186(61.2)	53(17.4)	65(21.4)

**Figure 1:**
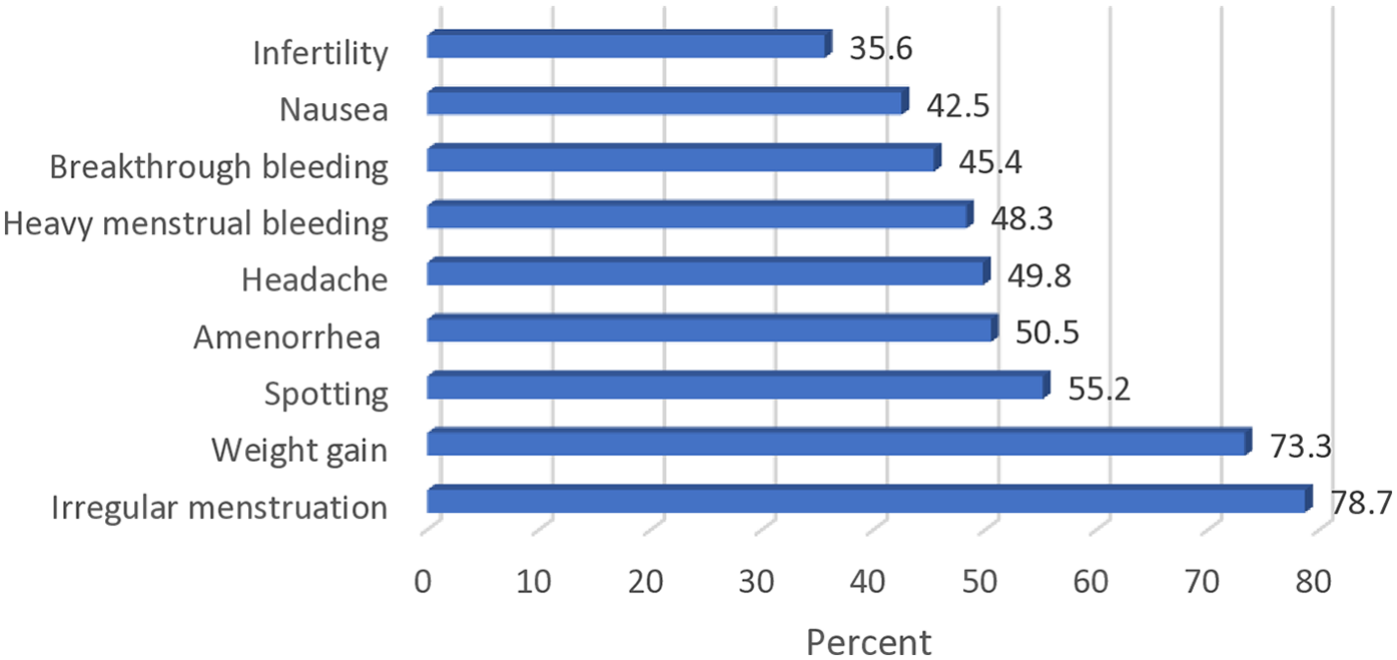
Common side effects of hormonal contraceptives.

As presented in Figure 1, the most frequently indicated side effect of contraceptives reported by the study participants was irregular menstruation (248/315, 78.7%), and this was closely followed by weight gain (231/315, 73.3%).

Also, from Figure 1, it can be seen that infertility was the least reported side effect (112/315, 35.6%) of hormonal contraceptives as indicated by the respondents, whilst half of the study participants (159/315, 50.5%) knew that the use of contraceptives could cause amenorrhea.

### Attitudes

The total mean score for the attitudes of the participants towards contraceptive use was 7.36 ± 2.40 (range; 1–10). Half of the participants (171/315, 56.6%) had a negative attitude towards contraception, a quarter of them (81/315, 25.7%) reported moderate attitudes, whilst only (63/315, 20.0%) of the respondents had positive attitudes in this regard.

More than half of the participants (169/297, 56.9%) were of the opinion that parental consent was not required by adolescents for contraceptive use, whilst a similar proportion (157/287, 54.7%) opined that providing contraceptives for this age group could promote promiscuity. The influence of religious and cultural beliefs is notable, as 56.7% cited religious opposition, and 39.6% align with cultural norms discouraging adolescent contraceptive use. Other relevant details are provided in Table 3.

**Table 3:** Attitudes towards contraceptives use.

SN	Statement	Agree	Disagree	Not sure
1	Unmarried adolescents do not require parental consent before contraceptives can be provided.	169 (56.9)	119 (40.1)	9 (2.9)
2	Unmarried adolescents should not be provided with contraceptives because it is wrong to engage in premarital sex.	140 (47.0)	149 (50.0)	9 (3.0)
3	Providing contraceptives for unmarried adolescents promotes sexual promiscuity.	157 (54.7)	112 (39.0)	18 (6.3)
4	It is better to tell sexually active unmarried adolescents to abstain from sex when they ask for contraceptives rather than give them contraceptives when they request for it.	150 (50.3)	133 (44.6)	15 (5.0)
5	Adolescents should be given contraceptive counselling before they become sexually active.	230 (79.0)	53 (18.2)	8 (2.7)
6	Healthcare providers should provide contraceptive services for both married and unmarried clients in healthcare facilities.	232 (78.4)	56 (18.9)	8 (2.5)
7	In my culture, it is wrong for adolescents to use contraceptives.	116 (39.6)	139 (47.4)	38 (13.0)
8	My religion does not allow the use of contraceptives by unmarried adolescents.	160 (56.7)	90 (31.9)	32 (11.3)
9	The benefits of family planning outweigh the risks.	226 (79.0)	44 (15.4)	16 (5.6)
10	Information about contraceptives should be included in sex education in secondary school.	228 (78.9)	46 (15.9)	15 (5.2)
11	Emergency oral contraceptives without prescription will promote unsafe sex.	180 (62.3)	93 (32.2)	16 (5.1)
12	It is important for all sexually active adults to be aware of emergency oral contraceptives.	241 (82.5)	37 (12.7)	14 (4.8)
13	Counselling of clients is important before recommending contraceptive pills.	262 (88.8)	23 (7.8)	10 (3.4)

### Practice

Almost all the participants (279/298, 93.6%) indicated recommending contraceptives to married adults, whilst only half of them (163/299, 54.5%) supported contraceptives' use for adolescents. A significant proportion of the participants (136/292, 46.1%) reported to have scolded adolescents who requested contraceptives. Further details on practice are provided in Table 4.

**Table 4:** Practice towards contraceptives use.

SN	Statement	Yes	No
1	Do you recommend contraceptives for married adults?	279 (93.6)	19 (6.4)
2	Do you recommend contraceptives for unmarried adolescents?	163 (54.5)	136 (45.5)
3	Do you recommend contraceptives for unmarried adults?	237 (80.3)	58 (19.7)
4	Do you counsel patients or clients about the likely hood of menstrual irregularities before dispensing or administering contraceptives to them?	231 (77.5)	67 (22.5)
5	Do you counsel on side effects of contraceptives?	244 (82.7)	51 (17.3)
6	Do you counsel on the time at which oral contraceptives should be taken?	222 (75.5)	72 (24.5)
7	I have scolded adolescents when they wanted contraceptives.	122 (41.8)	170 (58.2)
8	I have previously refused to recommend contraceptives to adolescents who are not yet married.	135 (46.1)	158 (53.9)
9	I limit my counselling on contraceptives to married persons only.	114 (39.6)	174 (60.4)

**Figure 2:**
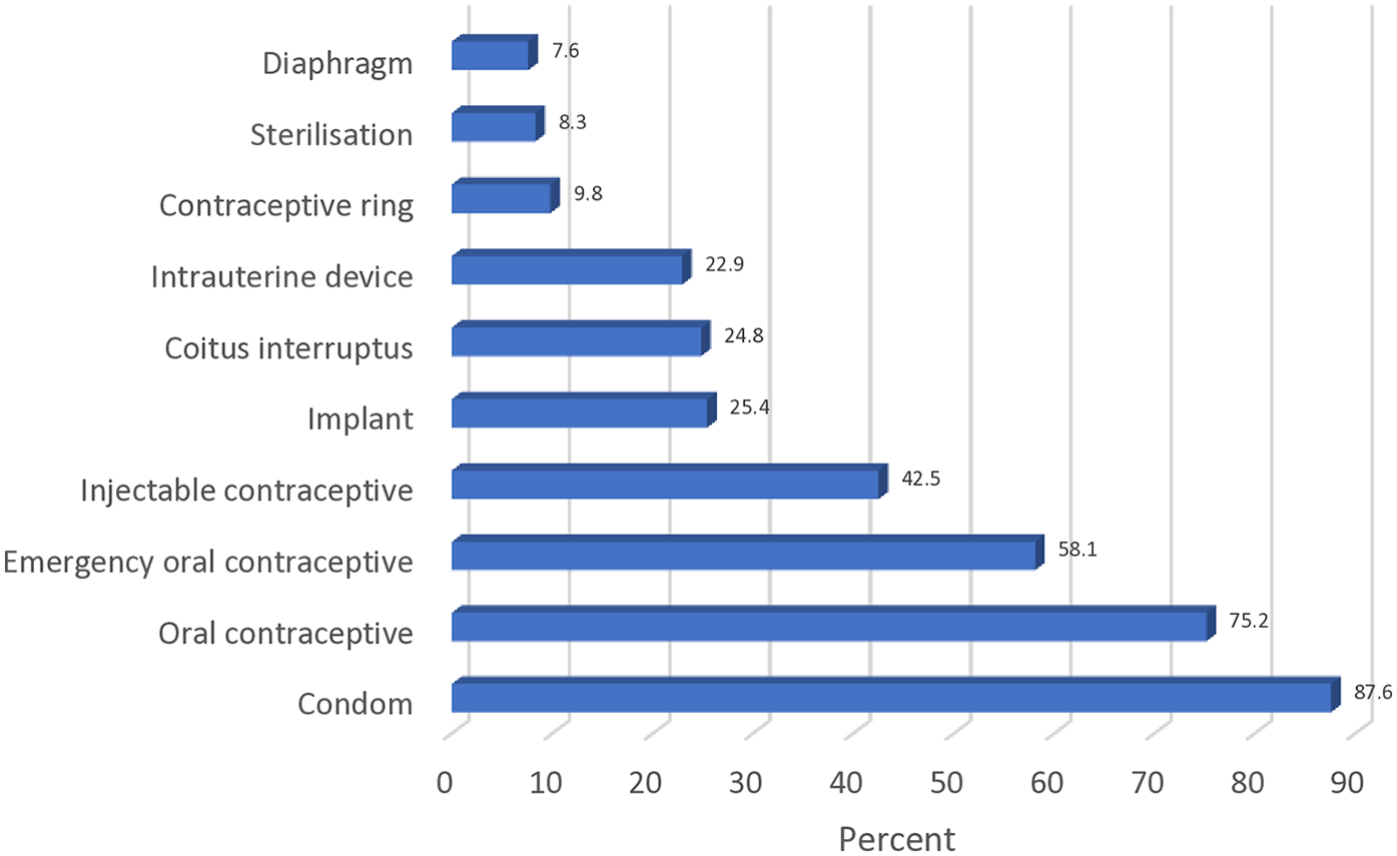
Methods of contraception promoted.

In terms of contraception methods promoted by the participants, condoms (276/315, 87.6%) emerged as the most commonly presented. This was closely followed by oral contraceptives (237/315, 75.2%). Further relevant details are provided in Figure 2.

Figure 2 shows the most common method of contraception promoted by the sampled community pharmacy staff. The least common methods of contraception were the diaphragm (24/315, 7.6%), sterilisation (26/315, 8.3%), and contraceptive ring (31/315, 9.8%). A quarter of the participants (78/315, 24.8%) indicated promoting coitus interruptus.

### Inferential statistical analysis of demography and knowledge

From the inferential statistical analysis undertaken, it was observed that the knowledge of the participants on contraceptive use was influenced by their socio-demographic characteristics. Male participants were more knowledgeable than the female respondents (*p* = 0.029), older participants had better knowledge (*p* = 0.001), and pharmacists reported a higher mean score compared to other groups of pharmacy staff (*p* < 0.001). Further details are provided in Table 5.

**Table 5:** Inferential statistical analysis of socio demographic characteristics and knowledge.

Variables	Category	Mean ± SD	Test of significance (*p*)
Gender			T = 2.196 (0.029)
Male		11.94 ± 3.42	
Female		11.12 ± 3.18	
Age			F = 5.005 (0.001)
<20	a	8.50 ± 3.45	
20–29	b	11.46 ± 3.44	
30–39	c	11.22 ± 3.19	
40–49	d	11.81 ± 3.64	
50 and above	e	13.56 ± 2.45	
Highest level of education			F = 3.304 (0.021)
Secondary education	a	10.71 ± 2.43	
Diploma/NCE	b	9.94 ± 3.27	
First degree/HND	c	11.91 ± 3.52	
Postgraduate level	d	11.52 ± 2.98	
Position & professional role			F = 13.827 (< 0.001)
Pharmacist	a	12.52 ± 3.23	
Pharmacy technician	b	10.31 ± 2.68	
Nurse	c	9.95 ± 2.84	
CHEW	d	9.93 ± 2.77	
Pharmacy support staff	e	9.37 ± 2.86	
Years of experience			F = 13.245 (< 0.001)
<5	a	11.96 ± 3.51	
5–10	b	10.49 ± 3.08	
>10	c	12.70 ± 3.00	

*Post hoc* tests.

Age: a vs. b (0.031); a vs. c (0.044); a vs. d (0.025); a vs. e (<0.001); b vs. c (0.581); b vs. d (0.627); b vs. e (0.001); c vs. d (0.402); c vs. e (< 0.001); d vs. e (0.039).

Highest level of education: a vs. b (0.572); a vs. c (0.346); a vs. d (0.531); b vs. c (0.002); b vs. d (0.018); c vs. d (0.325).

Position: a vs. b (0.006); a vs. c (< 0.001); a vs. d (< 0.001); a vs. e (<0.001); b vs. c (0.691); b vs. d (0.692); b vs. e (0.334); c vs. d (0.976); c vs. e (0.442); d vs. e (0.497).

Years of experience: a vs. b (0.001); a vs. c (0.120); b vs. c (< 0.001).

F = analysis of variance (ANOVA) test, t = student's *t*-test.

Additionally, multivariate linear regression was undertaken to determine multiple association. The R^2^ value was 0.21, indicating that 21% of the variance in the knowledge score was predicted by socio-demographic characteristics. The regression equation was significant (*F* = 12.11, *p* < 0.001) indicating that at least one of the six independent variables could significantly affect participants' knowledge. Participants' knowledge was influenced by age. Compared to those who were less than 20 years, respondents who were 20–29 years (*β* = 2.62; 95% CI = 0.07–5.17; *p* = 0.044), 30–39 (*β* = 2.85; 95% CI = 0.31–5.38; *p* = 0.028) as well as 50 and above (*β* = 3.18; 95% CI = 0.33–6.03; *p* = 0.029), were more knowledgeable and this was significant. Similarly, the position and professional role of the community pharmacy staff was another factor that affected knowledge. Pharmacists were significantly more knowledgeable (*β* = 2.85; 95% CI = 1.48–4.22; *p* = <0.001) than pharmacy support staff. Also, whilst pharmacy technicians, nurses and CHEW had more knowledge than the pharmacy support staff, this was not significant. Further details are presented in Table 6.

**Table 6:** Multivariable linear regression to determine the socio-demographic variables influencing knowledge.

Predictors	Unstandardised Coefficients		95% Confidence Interval (CI)	*p*-value
	B	Standard error		
Constant	6.93	1.80	3.40–10.47	<0.001
Gender				
Male	Reference category			
Female	−0.50	0.37	−1.22–0.22	0.174
Age				
<20	Reference category			
20–29	2.62	1.30	0.07–5.17	0.044
30–39	2.85	1.29	0.31–5.38	0.028
40–49	2.06	1.43	−0.76–4.88	0.152
50 and above	3.18	1.45	0.33–6.03	0.029
Highest level of education				
Secondary	Reference category			
Diploma/NCE	−0.53	1.30	−3.08–2.03	0.686
First degree	0.22	1.24	−2.21–2.66	0.856
Postgraduate level	−0.18	1.26	−2.59–2.35	0.925
Position & professional role				
Pharmacy support staff	Reference category			
Pharmacist	2.85	0.70	1.48–4.22	< 0.001
Pharmacy technician	1.06	0.97	−0.85–2.97	0.276
Nurse	0.83	0.78	−0.72–2.37	0.292
CHEW	0.64	0.86	−1.06–2.33	0.460
Years of Experience				
<5	Reference category			
5–10	−0.31	0.53	−1.35–0.74	0.567
>10	0.88	0.67	−0.43–2.20	0.187

## Discussion

This study provides novel insights regarding the knowledge, attitudes, and practices of community pharmacy staff towards contraception. Findings that emerged from this study suggest that the overall knowledge of the participants was suboptimal. However, the participants demonstrated adequate knowledge in certain aspects. For instance, a strong majority of the study cohort answered correctly that medical eligibility criteria for contraception provide guidance for practitioners regarding persons who should be considered fit to use contraceptive methods safely. The overall mean score for knowledge was just a little above average, suggesting a lack of comprehensive knowledge towards contraception. These findings are similar to other studies where an unsatisfactory level of knowledge was reported amongst pharmacists (5, 34).

Whilst findings from this study are consistent with international reports on the gaps in knowledge of contraceptives among pharmacy staff, the specific barriers identified in this study require context-specific solutions. Compared to studies in high-income countries, the respondents of this study exhibited concerns about providing contraception to unmarried adolescents, which may have been influenced by moral and cultural considerations (35–38). This underscores the need for targeted training and education to bridge these gaps. In contrast, studies from countries with more comprehensive adolescent contraceptives' policies, are associated with robust integration of such services within their primary healthcare system (39).

In this study, participants included various categories of staff working in the community pharmacy setting, such as pharmacists and technicians. Findings from this study indicate a critical need for regular training and capacity building of community pharmacy staff to ensure that they are adequately informed as regards contraception and various relevant areas related to sexual health. This novel finding is particularly important, considering that community pharmacies are usually the first port of call for minor ailments and healthcare services (40, 41). Values clarification and further training on adolescent sexual and reproductive health, ethical considerations in healthcare, and non-judgmental counselling are essential components to be incorporated in the training curriculum of healthcare practitioners. These recommendations are in line with the goals of Nigeria's National Family Planning Blueprint (2020–2024), which emphasises the importance of training healthcare providers to improve contraceptive knowledge and accessibility (42).

Participants in this study were familiar with the common side effects associated with the use of contraceptives, as more than three-quarters of them indicated irregular menstruation as one of these effects. Respondents' age and years of practice significantly influenced the level of knowledge exhibited by the participants, as older professionals and those with longer years of practice reported higher knowledge scores. This implies that relevant knowledge acquired increased, based on the duration of practice. Pharmacists also reported a higher knowledge score compared to other categories of community pharmacy staff, and this was expected considering their more robust training as regards provision of healthcare services (35).

Insights from this study reveal that community pharmacy staff play a significant role in either facilitating or obstructing adolescents' access to necessary healthcare, particularly in the area of contraception. Collectively, close to half of the participants reported moderate and positive attitudes towards contraceptive use, whilst the remaining half of the study cohort had negative attitudes. The participants were against providing contraceptives to unmarried adolescents as the majority of them felt this could promote sexual promiscuity. These findings are similar to those of studies amongst other categories of healthcare professionals in Nigeria, and those undertaken in other settings (36–38, 43). Available evidence suggests that adolescents avoid accessing public health facilities due to these negative attitudes, alongside their fear of stigmatisation (44, 45). Young individuals seeking to prevent unwanted pregnancies often face criticism from pharmacy staff when they are merely trying to access essential healthcare services. A considerable proportion of the participants felt that parental consent should be sought before providing contraceptives to people of young age whilst, in essence, there is no provision for this in Nigerian legislation. Currently, Nigeria's reproductive health policies are ambiguous regarding access to contraceptive by adolescents, particularly in community pharmacies, consequently creating a gap that needs be addressed through contextual reforms. For government and policymakers, this provides an opportunity to articulate a robust and comprehensive framework to guide healthcare workers in the discharge of their responsibilities as it relates to population access to contraceptives. Establishing such frameworks, can enable government address the confusion around adolescent contraceptives' access and ensure that healthcare providers, particularly in community pharmacies, have the necessary guidelines to serve young populations responsibly.

A strong majority of the participants demonstrated good practice in their outlook for the recommendation of contraceptives to both married and unmarried adults. However, a little less than half of the participants opposed providing adolescents with contraceptives. Whilst this may be attributable to issues relating to morality, the phenomenon is worthy of further study. Abstinence from sex represents the best strategy to prevent unwanted pregnancies amongst young people, however, professionals need to be better trained as regards contraceptive access for populations unable to abstain. This is important especially as policy guidelines regarding the provision of contraceptives to unmarried adolescents seem to remain unclear amongst healthcare professionals (36). This gap in policy guidance necessitates immediate attention from Nigerian authorities to clarify the role of healthcare providers in providing contraception to adolescents, ensuring that all healthcare workers are aligned with national goals.

The commonly recommended contraceptive method indicated by the study participants was the use of condoms, closely followed by the use of oral contraceptive pills. The least recommended were diaphragms, contraceptive rings, and sterilisation. The recommendation pattern may be attributable to the community pharmacy study setting which inadvertently influenced the choice of contraceptive methods easily dispensed within their premises. Further studies that build on these emergent findings also need to be undertaken in other settings, as well as amongst other healthcare professional groups.

Results from the multivariable linear regression analysis suggest that both age and professional role of the study participants significantly influenced their knowledge of contraception. This finding aligns with several studies (46–48), which also highlight age as a key factor influencing contraceptive knowledge. Additionally, the bivariate linear regression analysis indicates that as knowledge about contraception increases, attitudes toward contraception become more positive. This finding is consistent with a study by Dehlendorf and colleagues (49) which also emphasized the positive relationship between knowledge and attitudes towards contraception

A convenience sampling strategy was adopted to recruit participants, meaning that the sample may not have been representative of the population of community pharmacy staff in the Federal Capital Territory. However, this limitation was mitigated by various strengths of the study, which include validation and a robust pre-testing of the research instrument. Another limitation of this study is the potential for self-reporting bias, where participants may provide socially desirable answers or intentionally misrepresent their views to create a favourable impression. This weakness was minimized by employing the use of a validated questionnaire.

## Conclusion

This study reported poor knowledge and negativity in attitudes towards contraception. Although the overall knowledge of the participants was suboptimal, the participants demonstrated adequate knowledge in certain aspects, such as medical eligibility criteria for contraceptive use as well as the effect of cigarette smoking on some methods of contraception. Negative attitudes amongst healthcare providers can prevent people from accessing relevant services in the area of reproductive health. The participants appear to be familiar with the common side effects of contraceptives. Age and years of practice significantly influenced the level of knowledge exhibited by participants. Also, pharmacists reported a higher knowledge score compared to other categories of community pharmacy staff. Furthermore, the majority of the participants demonstrated good practice in their outlook for the recommendation of contraceptives to both married and unmarried adults. This study revealed that a little less than half of the participants opposed providing adolescents with contraceptives. The novel findings that emerged from this study can guide government and policymakers in developing relevant strategies that prevent unintended pregnancies amongst the populace.

Clear contextual policy guidelines can enable access to contraceptives for members of the public and prevent discriminative practice among healthcare professionals. Also, this is critically important to provide training for, and build the capacity of healthcare workers as regards service provision for the public in this area. As well as reducing unintended pregnancies, this intervention will also mitigate consequent health complications and associated socio-economic burden.

As this is the first study amongst community pharmacy staff in this setting, the novel findings that emerged provide an empirical basis for contextual policy and practice reforms which can be led by government and other relevant stakeholders. The development of relevant reproductive health guidelines that are fit for purpose in this context can enable improved access to contraceptives in community pharmacies and similar healthcare settings. Further studies can be undertaken to deepen the emergent findings from this study.

## Data Availability

The original contributions presented in the study are included in the article/Supplementary Material, further inquiries can be directed to the corresponding author/s.
