# Supplementary material for: Knowledge, attitudes, and practices regarding contraception amongst community pharmacy staff: a cross-sectional study in Nigeria
Source: Front Reprod Health. 2025 Mar 24;7:1488707. doi: 10.3389/frph.2025.1488707 (PMC11973348; doi:10.3389/frph.2025.1488707)
Supplement: Supplementary file 1 [file Table1.docx]

**Knowledge, attitudes, and practices towards contraception amongst community pharmacy staff**

**Introduction:**

Unintended pregnancy can occur due to failure to choose effective method of contraception or incorrect use of chosen method of contraception. This study aimed at assessing knowledge, attitude, and practice of contraceptive among community pharmacy staff. Please fill the questionnaire by ticking (√) the most appropriate option(s). Your responses will be treated confidentially.

**Demography**

1. Gender

| Male |  | Female |  |
| --- | --- | --- | --- |

1. Age

| **<** 20 |  | 20 - 29 |  | 30 – 39 |  | 40 – 49 |  | 50 and Above |  |
| --- | --- | --- | --- | --- | --- | --- | --- | --- | --- |

1. Educational Qualification

| Secondary School |  | Diploma/NCE |  | First Degree/HND |  | Postgraduate Level |  |
| --- | --- | --- | --- | --- | --- | --- | --- |

1. Position

| Pharmacist |  | Pharmacy Technician |  | Nurse |  | CHEW |  | Pharmacy Support Staff |  |
| --- | --- | --- | --- | --- | --- | --- | --- | --- | --- |

1. Years of Experience

| **<** 5 |  | 5 - 10 |  | >10 |  |
| --- | --- | --- | --- | --- | --- |

**Knowledge**

| **SN** | **Statement** | **True** | **False** | **I don’t know** |
| --- | --- | --- | --- | --- |
| 1 | Medical eligibility criteria for contraceptive use provides guidance regarding persons that can use contraceptive methods safely |  |  |  |
| 2 | Medical eligibility criteria for contraceptive use is only considered when the contraceptive to be administered is parenteral. |  |  |  |
| 3 | There could be delay in returning to full fertility after discontinuation of parenteral hormonal contraceptive. |  |  |  |
| 4 | In some cases, permanent infertility can occur as a result of hormonal contraceptive use. |  |  |  |
| 5 | Cigarette smoking can increase the risk of serious cardiovascular problems from combined oral contraceptive use. |  |  |  |
| 6 | Progestogens-only pills are less effective than combined pills. |  |  |  |
| 7 | Progestogens-only pills may be recommended when oestrogen is contraindicated. |  |  |  |
| 8 | Long term use of combined oral contraceptives is associated with reduced risk of endometrial and ovarian cancer. |  |  |  |
| 9 | Oral contraceptives belong to OTC medication. |  |  |  |
| 10 | Combined oral contraceptive is most effective if started at day 1 of menstrual cycle. |  |  |  |
| 11 | Emergency oral contraceptive when taken immediately after unprotected sexual intercourse has the same level of effectiveness when taken 72 hours after unprotected sexual intercourse. |  |  |  |
| 12 | Male condom should be removed while the penis is still erect |  |  |  |
| 13 | Spermicide is applied on the surface of the penis prior to intercourse |  |  |  |
| 14 | Compared to methods like IUDs, male condoms, and hormonal contraceptives, coitus interruptus method is more effective in preventing pregnancy |  |  |  |
| 15 | Emergency oral contraceptive works by preventing ovulation from occurring |  |  |  |
| 16 | Emergency oral contraceptive contain higher doses of hormone as compared to regular pills |  |  |  |
| 17 | Sterilization is a method of contraception that is easily reversible for both male and female |  |  |  |
| 18 | Contraception is an effective means for family planning |  |  |  |
| 19 | Vasectomy is the most effective permanent form of contraception available to men. |  |  |  |
| 20 | The procedure for vasectomy is minimally invasive |  |  |  |
| 21 | Tubal litigation is a female sterilization in which the fallopian tubes are permanently blocked or removed |  |  |  |

22. Common side effects of contraceptives (Tick all relevant options)

| a | Heavy menstrual bleeding |  |
| --- | --- | --- |
| b | Irregular menstruation |  |
| c | Headache |  |
| d | Spotting |  |
| e | Breakthrough bleeding |  |
| f | Nausea |  |
| g | Amenorrhea |  |
| h | Weight gain |  |
| i | Infertility |  |
| j | Others, please specify ........................................... | |

**Attitude**

| **SN** | **Statement** | **Agree** | **Disagree** | **Not Sure** |
| --- | --- | --- | --- | --- |
| 1 | Unmarried adolescents do not require parental consent before contraceptives can be provided. |  |  |  |
| 2 | Unmarried adolescents should not be provided with contraceptives because it is wrong to engage in premarital sex. |  |  |  |
| 3 | Providing contraceptives for unmarried adolescents promotes sexual promiscuity. |  |  |  |
| 4 | It is better to tell sexually active unmarried adolescents to abstain from sex when they ask for contraceptives rather than give them contraceptives when they request for it. |  |  |  |
| 5 | Adolescents should be given contraceptive counselling before they become sexually active. |  |  |  |
| 6 | Healthcare providers should provide contraceptive services for both married and unmarried clients in healthcare facilities. |  |  |  |
| 7 | In my culture, it is wrong for adolescents to use contraceptives. |  |  |  |
| 8 | My religion does not allow the use of contraceptives by unmarried adolescents. |  |  |  |
| 9 | The benefits of family planning outweigh the risks |  |  |  |
| 10 | Information about contraceptives should be included in sex education in secondary school. |  |  |  |
| 11 | Emergency oral contraceptives without prescription will promote unsafe sex. |  |  |  |
| 12 | It is important for all sexually active adults to be aware of emergency oral contraceptive. |  |  |  |
| 13 | Counselling of clients is important before recommending contraceptive pills. |  |  |  |

**Practice**

| **SN** | **Statement** | **Yes** | **No** |
| --- | --- | --- | --- |
| 1 | Do you recommend contraceptives for married adults? |  |  |
| 2 | Do you recommend contraceptives for unmarried adolescents? |  |  |
| 3 | Do you recommend contraceptives for unmarried adults? |  |  |
| 4 | Do you counsel patients or clients about the likely hood of menstrual irregularities before dispensing or administering contraceptives to them? |  |  |
| 5 | Do you counsel on side effects of contraceptives? |  |  |
| 6 | Do you counsel on the time at which oral contraceptives should be taken? |  |  |
| 7 | I have scolded adolescents when they wanted contraceptives. |  |  |
| 8 | I have previously refused to recommend contraceptives to adolescents who are not yet married. |  |  |
| 9 | I limit my counselling on contraceptives to married persons only. |  |  |

11. Which of these methods of contraception have you promoted in the past? (tick all the relevant options)

| a | Condom |  |
| --- | --- | --- |
| b | Oral contraceptive pill |  |
| c | Intrauterine device |  |
| d | Contraceptive implant |  |
| e | Contraceptive injection |  |
| f | Emergency contraceptive pill |  |
| g | Contraceptive ring |  |
| h | Diaphragm |  |
| i | Sterilization (vasectomy or tubal litigation) |  |
| j | Coitus interruptus |  |
