# Supplementary material for: Knowledge, attitudes, and practices regarding contraception amongst community pharmacy staff: a cross-sectional study in Nigeria
Source: Front Reprod Health. 2025 Mar 24;7:1488707. doi: 10.3389/frph.2025.1488707 (PMC11973348; doi:10.3389/frph.2025.1488707)
Supplement: Supplementary file 2 [file Table2.docx]

**Knowledge, attitudes, and practices towards contraception amongst community pharmacy staff: a cross-sectional study in Nigeria**

Obi Peter Adigwe^1^, Godspower Onavbavba^1^

^1^Affiliation: National Institute for Pharmaceutical Research and Development

Address: Plot 942, Cadastral Zone C16, Idu Industrial District, Abuja, Federal Capital Territory, Nigeria.

**Authors’ Email addresses**

Obi Peter Adigwe: [o.p.adigwe@niprd.gov.ng](mailto:o.p.adigwe@niprd.gov.ng)

Godspower Onavbavba: [onavbavbagodspower@gmail.com](mailto:onavbavbagodspower@gmail.com)

**ORCID details**

Obi Peter Adigwe: <https://orcid.org/0000-0001-8832-6459>

Godspower Onavbavba: <https://orcid.org/0000-0002-4803-8804>

**Correspondence**

Name: Obi Peter Adigwe

Email: [o.p.adigwe@niprd.gov.ng](mailto:o.p.adigwe@niprd.gov.ng)

Address: National Institute for Pharmaceutical Research and Development, Plot 942, Cadastral Zone C16, Idu Industrial District, Abuja, Federal Capital Territory, Nigeria.

**Abstract**

**Introduction:** Lack of access to effective methods of contraception can increase the prevalence of unintended pregnancies, alongside possible deleterious health consequences. Community pharmacies represent essential points for population access to contraceptives. They have also been identified as a useful resource in supporting counselling services to the public. This study aimed at assessing the knowledge, attitudes, and practices of community pharmacy staff towards contraception.

**Methods:** A cross-sectional study was undertaken in the Federal Capital Territory, Nigeria. Data were collected from 315 community pharmacy staff using self-administered questionnaires. The participants’ knowledge and attitude scores were categorised using Bloom’s cut-off point. Analyses were undertaken using Statistical Package for Social Sciences. The data were analysed using frequency distribution, chi-square, and linear regression at a 5% level of significance.

**Results:** Male participants in the study (165/315, 52.4%) were slightly higher than the female respondents (150/315, 47.6%), and about two-thirds of the study cohort were pharmacists (200/315, 63.5%). The majority of the participants (183/315, 58.1%) had poor knowledge of contraceptives use. A quarter of them (81/315, 25.7%) reported moderate attitudes. Almost all the participants (279/298, 93.6%) indicated recommending contraceptives for married adults, and a significant proportion of them (136/292, 45.5%) were opposed to recommending contraceptives for unmarried adolescents. Knowledge was significantly influenced by age (*p=*0.001). Also, position was also seen to significantly influence the knowledge of the study respondents towards contraceptives (p<0.001)

**Conclusion:** Findings from this study revealed poor knowledge and negative attitudes of community pharmacy staff towards contraception. Government and relevant stakeholders can build on these novel findings in reforming pertinent contextual policies and practices. This can significantly improve access to contraceptives amongst the populace, and consequently reduce unintended pregnancies alongside possible health and societal implications.

**Keywords:** Pregnancy, abortion, contraceptives, birth control, family planning.

**Introduction**

Globally, an estimated 250 million pregnancies occur each year, with up to one-third of these pregnancies being unintended [1,2]. Unintended refers to unwanted or mistimed pregnancies which can occur due to failure to choose an effective contraception method, or incorrect use of contraceptives [3,4]. Such pregnancies can place an economic burden on societies, as well as lead to reproductive health risks for women [5]. Close to a quarter of unwanted pregnancies are terminated using unsafe methods, and up to 18% end up in unplanned births [6]. Whilst unsafe termination of pregnancies is common in low-income countries, available evidence suggests that the abortion rates in developing and developed countries are of a similar proportion [7]. In Nigeria, between 2015 and 2019, 10.5 million pregnancies occur annually, with 2.99 million being unintended, and 1.43 million ending in abortion [7]. This high abortion rates and safety concerns in low-income countries highlight the need for improved access to contraception, especially from frontline healthcare practitioners in the community pharmacy practice.

Contraception, also known as birth control, anticonception, and fertility control, refers to various relevant methods used for the prevention of pregnancies. Contraception has been in use for a long time; however, effective and safe methods of birth control only became available in the 20^th^ century [8]. Methods such as sterilisation, the use of intrauterine devices, and implantable birth control have been identified as the most effective means of fertility control, and this list is followed by other methods such as oral pills, patches, vaginal rings, and injections [9]. Other techniques that are categorised as less effective include barrier methods, such as the use of condoms, diaphragm, and birth control sponges. The least effective birth control methods are the use of spermicides and withdrawal methods. Pregnancies in teenagers are associated with a greater risk of poor outcomes [10]. Promoting comprehensive sex education and access to contraceptives for this age group decreases the rate of unwanted pregnancies [11]. Birth control can improve women’s delivery outcomes and the survival of their children by increasing the length of time between pregnancies [12].

In Nigeria, contraceptive use is shaped by a complex interplay of cultural and systemic factors [13]. Deeply rooted religious beliefs and societal stigma often create significant barriers, discouraging open discussions about contraception and complicating access for individuals seeking options [14]. These challenges are further exacerbated by gender dynamics, where family planning decisions are frequently dominated by men, limiting women's autonomy in making informed choices [15].

The public can experience barriers in accessing contraceptives, especially when some health providers feel that certain individuals that are unmarried should not receive such services due to their personal belief. These barriers can manifest in various forms, including biased attitudes, lack of comprehensive training on reproductive health, and inadequate communication about contraceptive options [16]. Providers may perpetuate stigma by questioning the moral or social appropriateness of contraceptive use among certain populations, leading to discriminatory practices that deny essential services [17]. Additionally, the insufficient integration of family planning education into healthcare training programs means that many practitioners lack the knowledge necessary to provide unbiased, evidence-based information about contraceptives [18]. As a result, these barriers not only limit access to contraceptives but also undermine broader public health efforts aimed at reducing unintended pregnancies and promoting reproductive rights in Nigeria.

The community pharmacy setting represents an essential point for accessing contraceptives. This setting also serves as a venue for offering counselling services to the public as well as a first point of call for issues relating to birth control pills [19-21]. Community pharmacies play a pivotal role in access to contraceptives, and this can be influenced by the knowledge and attitudes of the healthcare personnel practicing in this setting. Appropriate knowledge, positive attitudes, and good practices are critical elements that can enable a community pharmacy staff to provide comprehensive counselling, as well as create necessary awareness regarding contraceptives, thus preventing unintended pregnancies [22,23]. Several studies have been undertaken in relation to knowledge, attitudes, and practices towards contraceptive use in Nigeria [24,25], there is however paucity of information about the community pharmacy staff in this area. It is against this backdrop that this study aimed at assessing the knowledge, attitudes, and practices of community pharmacy staff towards contraception.

**Methods**

**Study design**

The study was undertaken between May and August 2022 in Nigeria’s Federal Capital Territory using a cross-sectional study design. The data collection tool (supplementary file) was developed following an extensive literature review [5, 26-29]. The items in the instrument were knowledge, attitudes, and practices towards contraceptives, as well as a section on socio-demographic characteristics. The study tool was structured to assess community pharmacy staff in these thematic areas. The items assessing knowledge were answered on a “true/false” basis and an additional “I do not know” option. The questions assessing attitudes were structured as “agree”, “disagree”, and “not sure”, whilst practice questions were answered on a “yes” or “no” basis.

**Validation of research instrument**

Questionnaire validation was undertaken by an expert panel comprising faculty members engaged in research activities in the field of contraception. These experts were chosen from various institutions, and were made of individuals who are familiar with survey design, validation processes, research methodologies as well as psychometrics and construct validity. Face and content validations were undertaken. The study tool was assessed for appropriateness, complexity, attractiveness, and relevance of the items. Content validity ratio and content validity index tests were undertaken for each item, and only those that passed these tests were included in the questionnaire. Cronbach alpha’s test was also conducted to assess the reliability of the questionnaire and this gave a value of 0.83, indicating internal consistency in the questionnaire items. The questionnaire was pilot-tested by administering it to an initial cohort of 20 participants who were randomly selected. The feedback received did not necessitate any further change, and this led to the final version of the questionnaire.

**Sampling**

According to a study by Ekpenyong *et al.* [30], there are 455 registered community pharmacies in the Federal Capital Territory. A minimum sample size of 314 was calculated for an estimated number of 1700 pharmacists and pharmacy support staff in the Federal Capital Territory. This was computed at 95% confidence level, 5% margin of error, and 50% response distribution using Epi Info software version 7 [31]. This is in tandem with Cochran’s formula for calculating the sample size of a finite population [32]. The sample size was rounded up to 400 to account for non-response. Participants were recruited following a convenience sampling strategy deployed across community pharmacies in the Federal Capital Territory to get enough respondents. Inclusion criteria adopted for the study include pharmacists licensed to practice; trained pharmacy technicians; and support staff involved in roles pertaining to dispensing. Pharmacy staff who did not have any role to play in relation to dispensing medications to patients were excluded from the study. Paper-based questionnaires were administered to the study participants.

**Ethics consideration**

Prior to the data collection phase, ethical approval was obtained from the Federal Capital Territory Health Research Ethics Committee (Approval number: FHREC/2021/01/97/12-08-21), and participation in the study was voluntary. Written informed consent was obtained from the participants before administering the questionnaires. Confidentiality and anonymity were strictly maintained throughout the data collection process. All information that could link participants to their responses was not included in the data collection tool.

**Data analysis**

Following the retrieval of questionnaires, data were entered into Statistical Package for Social Sciences version 25. Descriptive statistical analyses were undertaken. For the knowledge section, each correct response was assigned a score of 1, and incorrect responses and unanswered questions were assigned 0. The participants’ overall knowledge score was categorised using Bloom’s cut-off point as good for 80-100%, moderate for 60-79%, and poor for ≤59% [33]. Bloom’s cut-off point was chosen because it provides a widely accepted framework for interpreting survey scores, ensuring consistency and comparability across studies on knowledge assessment. For the attitude section, questions were assigned 1 point for a positive attitude towards contraception, and 0 was assigned for unanswered or negative feedback. The total attitude score for each participant was categorised by also using Bloom’s cut-off as positive for 80-100%, moderate for 60-79%, and negative for ≤59%. Findings from the practice section were presented as percentages and frequencies as this approach is more appropriate for capturing behavioral trends.

Student’s t-test and analysis of variance (ANOVA) were undertaken to determine relationships between mean knowledge scores and socio-demographic characteristics. Post hoc analysis (LSD) was performed in cases of significant ANOVA tests for multiple comparisons. A *p*-value of 0.05 or less represented the threshold for statistical significance. Linear regression was used with 95% confidence interval to show the strengths of association. Finally, a *p-*value of less than 0.05 in the multivariate regression analysis was used to identify variables significantly associated with the knowledge of contraception amongst community pharmacy staff.

**Results**

**Demography**

A total of 315 community pharmacy staff comprising (165/315, 52.4%) male participants and (150/315, 47.6%) female respondents participated in the study. Close to half of the sample (149/315, 47.3%) were between the ages of 30 to 39 years. Slightly above a third of the participants (114/315, 36.2%) were educated up to postgraduate level. Further details on the socio-demographic characteristics of the respondents are provided in Table 1.

**Table 1: Socio-demographic characteristics**

| **Variable** | **Frequency (%)** |
| --- | --- |
| Gender |  |
| Male | 165 (52.4) |
| Female | 150 (47.6) |
| Age |  |
| < 20 | 6 (1.9) |
| 20 – 29 | 100 (31.7) |
| 30 – 39 | 149 (47.3) |
| 40 – 49 | 26 (8.3) |
| 50 and above | 34 (10.8) |
| Highest level of education |  |
| Secondary education | 7 (2.2) |
| National diploma/NCE | 31 (9.8) |
| First degree | 163 (51.7) |
| Postgraduate | 114 (36.2) |
| Position |  |
| Pharmacist | 200 (63.5) |
| Pharmacy technician | 16 (5.1) |
| Nurse | 43 (13.7) |
| CHEW | 29 (9.2) |
| Pharmacy support staff | 27 (8.6) |
| Years of experience |  |
| < 5 | 102 (32.4) |
| 5 – 10 | 130 (41.3) |
| > 10 | 83 (26.3) |

**Knowledge**

The total mean knowledge score for the participants was 11.55 ± 3.33 (range; 1 – 21). The majority of the participants (183/315, 58.1%) had poor knowledge regarding contraception, a third of the participants (110/315, 34.9%) reported moderate knowledge, whilst only a few of the respondents (22/315, 7.0%) had good knowledge.

In terms of medical eligibility criteria for contraceptive use, a strong majority of the participants (273/315, 86.7%) indicated correctly that the eligibility criteria provides guidance regarding which clients can use contraception methods safely. About three-quarters (231/315, 73.3%) of the participants knew that cigarette smoking could increase the risk of serious cardiovascular problems from combined oral contraceptive use. Only a third of the participants were fully knowledgeable about hormonal contraceptives’ lack of association (108/315, 34.3%) with permanent infertility.

**Table 2: knowledge towards use of contraceptives**

| **SN** | **Statement** | **True** | **False** | **I don’t know** |
| --- | --- | --- | --- | --- |
| 1 | Medical eligibility criteria for contraceptive use provides guidance regarding persons that can use contraceptive methods safely | 273(87.5) | 26(8.3) | 13(4.2) |
| 2 | Medical eligibility criteria for contraceptive use is only considered when the contraceptive to be administered is parenteral. | 106(34.4) | 180(58.4) | 22(7.1) |
| 3 | There could be delay in returning to full fertility after discontinuation of parenteral hormonal contraceptive. | 216(70.6) | 71(23.2) | 19(6.2) |
| 4 | In some cases, permanent infertility can occur as a result of hormonal contraceptive use. | 151(50.0) | 108(35.8) | 43(14.2) |
| 5 | Cigarette smoking can increase the risk of serious cardiovascular problems from combined oral contraceptive use. | 231(75.2) | 39(12.7) | 37(12.1) |
| 6 | Progestogens-only pills are less effective than combined pills. | 157(51.6) | 102(33.6) | 45(14.8) |
| 7 | Progestogens-only pills may be recommended when oestrogen is contraindicated. | 196(65.1) | 53(17.6) | 52(17.3) |
| 8 | Long term use of combined oral contraceptives is associated with reduced risk of endometrial and ovarian cancer. | 146(48.8) | 100(33.4) | 53(17.7) |
| 9 | Oral contraceptives belong to OTC medication. | 181(60.7) | 93(31.2) | 24(8.1) |
| 10 | Combined oral contraceptive is most effective if started at day 1 of menstrual cycle. | 180(61.6) | 67(22.9) | 45(15.4) |
| 11 | Emergency oral contraceptive when taken immediately after unprotected sexual intercourse has the same level of effectiveness when taken 72 hours after unprotected sexual intercourse. | 127(41.1) | 159(51.5) | 23(7.4) |
| 12 | Male condom should be removed while the penis is still erect | 160(53.3) | 92(30.7) | 48(16.0) |
| 13 | Spermicide is applied on the surface of the penis prior to intercourse | 146(49.0) | 96(32.2) | 56(18.8) |
| 14 | Compared to methods like IUDs, male condoms, and hormonal contraceptives, coitus interruptus method is more effective in preventing pregnancy | 131(43.1) | 145(47.7) | 28(9.2) |
| 15 | Emergency oral contraceptive works by preventing ovulation from occurring | 131(44.3) | 140(47.3) | 25(8.4) |
| 16 | Emergency oral contraceptive contain higher doses of hormone as compared to regular pills | 167(56.0) | 84(28.2) | 46(15.4) |
| 17 | Sterilization is a method of contraception that is easily reversible for both male and female | 109(36.3) | 162(54.0) | 29(9.7) |
| 18 | Contraception is an effective means for family planning | 231(75.7) | 59(19.3) | 15(4.9) |
| 19 | Vasectomy is the most effective permanent form of contraception available to men. | 193(62.9) | 71(23.1) | 43(14.0) |
| 20 | The procedure for vasectomy is minimally invasive | 131(43.4) | 79(26.2) | 92(30.5) |
| 21 | Tubal litigation is a female sterilization in which the fallopian tubes are permanently blocked or removed | 186(61.2) | 53(17.4) | 65(21.4) |

As presented in Figure 1, the most frequently indicated side effect of contraceptives reported by the study participants was irregular menstruation (248/315, 78.7%), and this was closely followed by weight gain (231/315, 73.3%).

**Figure 1: Common side effects of hormonal contraceptives**

Also, from Figure 1, it can be seen that infertility was the least reported side effect (112/315, 35.6%) of hormonal contraceptives as indicated by the respondents, whilst half of the study participants (159/315, 50.5%) knew that the use of contraceptives could cause amenorrhea.

**Attitudes**

The total mean score for the attitudes of the participants towards contraceptive use was 7.36 ± 2.40 (range; 1 – 10). Half of the participants (171/315, 56.6%) had a negative attitude towards contraception, a quarter of them (81/315, 25.7%) reported moderate attitudes, whilst only (63/315, 20.0%) of the respondents had positive attitudes in this regard.

More than half of the participants (169/297, 56.9%) were of the opinion that parental consent was not required by adolescents for contraceptive use, whilst a similar proportion (157/287, 54.7%) opined that providing contraceptives for this age group could promote promiscuity. The influence of religious and cultural beliefs is notable, as 56.7% cited religious opposition, and 39.6% align with cultural norms discouraging adolescent contraceptive use. Other relevant details are provided in Table 3.

**Table 3: Attitudes towards contraceptives use**

| **SN** | **Statement** | **Agree** | **Disagree** | **Not Sure** |
| --- | --- | --- | --- | --- |
| 1 | Unmarried adolescents do not require parental consent before contraceptives can be provided. | 169 (56.9) | 119 (40.1) | 9 (2.9) |
| 2 | Unmarried adolescents should not be provided with contraceptives because it is wrong to engage in premarital sex. | 140 (47.0) | 149 (50.0) | 9 (3.0) |
| 3 | Providing contraceptives for unmarried adolescents promotes sexual promiscuity. | 157 (54.7) | 112 (39.0) | 18 (6.3) |
| 4 | It is better to tell sexually active unmarried adolescents to abstain from sex when they ask for contraceptives rather than give them contraceptives when they request for it. | 150 (50.3) | 133 (44.6) | 15 (5.0) |
| 5 | Adolescents should be given contraceptive counselling before they become sexually active. | 230 (79.0) | 53 (18.2) | 8 (2.7) |
| 6 | Healthcare providers should provide contraceptive services for both married and unmarried clients in healthcare facilities. | 232 (78.4) | 56 (18.9) | 8 (2.5) |
| 7 | In my culture, it is wrong for adolescents to use contraceptives. | 116 (39.6) | 139 (47.4) | 38 (13.0) |
| 8 | My religion does not allow the use of contraceptives by unmarried adolescents. | 160 (56.7) | 90 (31.9) | 32 (11.3) |
| 9 | The benefits of family planning outweigh the risks. | 226 (79.0) | 44 (15.4) | 16 (5.6) |
| 10 | Information about contraceptives should be included in sex education in secondary school. | 228 (78.9) | 46 (15.9) | 15 (5.2) |
| 11 | Emergency oral contraceptives without prescription will promote unsafe sex. | 180 (62.3) | 93 (32.2) | 16 (5.1) |
| 12 | It is important for all sexually active adults to be aware of emergency oral contraceptive. | 241 (82.5) | 37 (12.7) | 14 (4.8) |
| 13 | Counselling of clients is important before recommending contraceptive pills. | 262 (88.8) | 23 (7.8) | 10 (3.4) |

**Practice**

Almost all the participants (279/298, 93.6%) indicated recommending contraceptives to married adults, whilst only half of them (163/299, 54.5%) supported contraceptives’ use for adolescents. A significant proportion of the participants (136/292, 46.1%) reported to have scolded adolescents who requested contraceptives. Further details on practice are provided in Table 4.

**Table 4: Practice towards contraceptives use**

| **SN** | **Statement** | **Yes** | **No** |
| --- | --- | --- | --- |
| 1 | Do you recommend contraceptives for married adults? | 279 (93.6) | 19 (6.4) |
| 2 | Do you recommend contraceptives for unmarried adolescents? | 163 (54.5) | 136 (45.5) |
| 3 | Do you recommend contraceptives for unmarried adults? | 237 (80.3) | 58 (19.7) |
| 4 | Do you counsel patients or clients about the likely hood of menstrual irregularities before dispensing or administering contraceptives to them? | 231 (77.5) | 67 (22.5) |
| 5 | Do you counsel on side effects of contraceptives? | 244 (82.7) | 51 (17.3) |
| 6 | Do you counsel on the time at which oral contraceptives should be taken? | 222 (75.5) | 72 (24.5) |
| 7 | I have scolded adolescents when they wanted contraceptives. | 122 (41.8) | 170 (58.2) |
| 8 | I have previously refused to recommend contraceptives to adolescents who are not yet married. | 135 (46.1) | 158 (53.9) |
| 9 | I limit my counselling on contraceptives to married persons only. | 114 (39.6) | 174 (60.4) |

In terms of contraception methods promoted by the participants, condoms (276/315, 87.6%) emerged as the most commonly presented. This was closely followed by oral contraceptives (237/315, 75.2%). Further relevant details are provided in Figure 2.

**Figure 2: Methods of contraception promoted**

Fig 2 shows the most common method of contraception promoted by the sampled community pharmacy staff. The least common methods of contraception were the diaphragm (24/315, 7.6%), sterilisation (26/315, 8.3%), and contraceptive ring (31/315, 9.8%). A quarter of the participants (78/315, 24.8%) indicated promoting coitus interruptus.

**Inferential statistical analysis of demography and knowledge**

From the inferential statistical analysis undertaken, it was observed that the knowledge of the participants on contraceptive use was influenced by their socio-demographic characteristics. Male participants were more knowledgeable than the female respondents (*p* = 0.029), older participants had better knowledge (*p* = 0.001), and pharmacists reported a higher mean score compared to other groups of pharmacy staff (*p* < 0.001). Further details are provided in Table 5.

**Table 5: Inferential statistical analysis of socio demographic characteristics and knowledge**

| **Variables** | **Category** | **Mean ± SD** | **Test of Significance (*p*)** |
| --- | --- | --- | --- |
| Gender |  |  | T = 2.196 (0.029) |
| Male |  | 11.94 ± 3.42 |  |
| Female |  | 11.12 ± 3.18 |  |
| Age |  |  | F = 5.005 (0.001) |
| < 20 | a | 8.50 ± 3.45 |  |
| 20-29 | b | 11.46 ± 3.44 |  |
| 30-39 | c | 11.22 ± 3.19 |  |
| 40-49 | d | 11.81 ± 3.64 |  |
| 50 and above | e | 13.56 ± 2.45 |  |
| Highest level of education |  |  | F = 3.304 (0.021) |
| Secondary education | a | 10.71 ± 2.43 |  |
| Diploma/NCE | b | 9.94 ± 3.27 |  |
| First degree/HND | c | 11.91 ± 3.52 |  |
| Postgraduate level | d | 11.52 ± 2.98 |  |
| Position |  |  | F = 13.827 (< 0.001) |
| Pharmacist | a | 12.52 ± 3.23 |  |
| Pharmacy technician | b | 10.31 ± 2.68 |  |
| Nurse | c | 9.95 ± 2.84 |  |
| CHEW | d | 9.93 ± 2.77 |  |
| Pharmacy support staff | e | 9.37 ± 2.86 |  |
| Years of experience |  |  | F = 13.245 (< 0.001) |
| < 5 | a | 11.96 ± 3.51 |  |
| 5 – 10 | b | 10.49 ± 3.08 |  |
| > 10 | c | 12.70 ± 3.00 |  |

Post hoc tests

Age: a vs b (0.031); a vs c (0.044); a vs d (0.025); a vs e (< 0.001); b vs c (0.581); b vs d (0.627); b vs e (0.001); c vs d (0.402); c vs e (< 0.001); d vs e (0.039).

Highest level of education: a vs b (0.572); a vs c (0.346); a vs d (0.531); b vs c (0.002); b vs d (0.018); c vs d (0.325).

Position: a vs b (0.006); a vs c (< 0.001); a vs d (< 0.001); a vs e (< 0.001); b vs c (0.691); b vs d (0.692); b vs e (0.334); c vs d (0.976); c vs e (0.442); d vs e (0.497).

Years of experience: a vs b (0.001); a vs c (0.120); b vs c (< 0.001)

Note: F = analysis of variance (ANOVA) test, t = student’s t-test.

Additionally, multivariate linear regression was undertaken to determine multiple association . The R^2^ value was 0.21, indicating that 21% of the variance in the knowledge score was predicted by socio-demographic characteristics . The regression equation was significant (*F* = 12.11, *p* < 0.001) indicating that at least one of the six independent variables could significantly affect participants’ knowledge. Participants' knowledge was influenced by age. Compared to those who were less than 20 years, respondents who were 20 – 29 years (β = 2.62; 95% CI = 0.07 – 5.17; *p* = 0.044), 30 – 39 (β = 2.85; 95% CI = 0.31 – 5.38; *p* = 0.028) as well as 50 and above (β = 3.18; 95% CI = 0.33 – 6.03; *p* = 0.029), were more knowledgeable and this was significant. Similarly, the position of the community pharmacy staff was another factor that affected knowledge. Pharmacists were significantly more knowledgeable (β = 2.85; 95% CI = 1.48 – 4.22; *p* = < 0.001) than pharmacy support staff. Also, whilst pharmacy technicians, nurses and CHEW had more knowledge than the pharmacy support staff, this was not significant. Further details are presented in Table 6.

**Table 6: Multivariable Linear Regression to Determine the Socio-demographic Variables Influencing Knowledge**

| **Predictors** | **Unstandardised Coefficients** | | **95% Confidence Interval**  **(CI)** | ***p*-value** |
| --- | --- | --- | --- | --- |
|  | **B** | **Standard Error** |  |  |
| Constant | 6.93 | 1.80 | 3.40 – 10.47 | <0.001 |
| Gender |  |  |  |  |
| Male | Reference category | | | |
| Female | - 0.50 | 0.37 | - 1.22 – 0.22 | 0.174 |
| Age |  |  |  |  |
| <20 | Reference category | | | |
| 20 – 29 | 2.62 | 1.30 | 0.07 – 5.17 | 0.044 |
| 30 – 39 | 2.85 | 1.29 | 0.31 – 5.38 | 0.028 |
| 40 – 49 | 2.06 | 1.43 | - 0.76 – 4.88 | 0.152 |
| 50 and above | 3.18 | 1.45 | 0.33 – 6.03 | 0.029 |
| Highest level of education |  |  |  |  |
| Secondary | Reference category | | | |
| Diploma/NCE | - 0.53 | 1.30 | - 3.08 – 2.03 | 0.686 |
| First degree | 0.22 | 1.24 | - 2.21 – 2.66 | 0.856 |
| Postgraduate level | -0.18 | 1.26 | - 2.59 – 2.35 | 0.925 |
| Position |  |  |  |  |
| Pharmacy support staff | Reference category | | | |
| Pharmacist | 2.85 | 0.70 | 1.48 – 4.22 | < 0.001 |
| Pharmacy technician | 1.06 | 0.97 | - 0.85 – 2.97 | 0.276 |
| Nurse | 0.83 | 0.78 | - 0.72 – 2.37 | 0.292 |
| CHEW | 0.64 | 0.86 | - 1.06 – 2.33 | 0.460 |
| Years of Experience |  |  |  |  |
| < 5 | Reference category | | | |
| 5 – 10 | - 0.31 | 0.53 | - 1.35 – 0.74 | 0.567 |
| >10 | 0.88 | 0.67 | - 0.43 – 2.20 | 0.187 |

**Discussion**

This study provides novel insights regarding the knowledge, attitudes, and practices of community pharmacy staff towards contraception. Findings that emerged from this study suggest that the overall knowledge of the participants was suboptimal. However, the participants demonstrated adequate knowledge in certain aspects. For instance, a strong majority of the study cohort answered correctly that medical eligibility criteria for contraception provide guidance for practitioners regarding persons who should be considered fit to use contraceptive methods safely. The overall mean score for knowledge was just a little above average, suggesting a lack of comprehensive knowledge towards contraception. These findings are similar to other studies where an unsatisfactory level of knowledge was reported amongst pharmacists [5,34].

Whilst findings from this study are consistent with international reports on the gaps in knowledge of contraceptives among pharmacy staff, the specific barriers identified in this study require context-specific solutions. Compared to studies in high-income countries, the respondents of this study exhibited concerns about providing contraception to unmarried adolescents, which may have been influenced by moral and cultural considerations [35-38]. This underscores the need for targeted training and education to bridge these gaps. In contrast, studies from countries with more comprehensive adolescent contraceptives’ policies, are associated with robust integration of such services within their primary healthcare system [39].

In this current study, participants were made up of different categories of staff working in the community pharmacy setting, including pharmacists. Findings from this study indicate a critical need for regular training and capacity building of community pharmacy staff to ensure that they are adequately informed as regards contraception and various relevant areas related to sexual health.. This novel finding is particularly important, considering that community pharmacies are usually the first port of call for minor ailments and healthcare services [40,41]. Values clarification and further training on adolescent sexual and reproductive health, ethical considerations in healthcare, and non-judgmental counselling are essential components to be incorporated in the training curriculum of healthcare practitioners. These recommendations are in line with the goals of Nigeria’s National Family Planning Blueprint (2020–2024), which emphasises the importance of training healthcare providers to improve contraceptive knowledge and accessibility [42].

Participants of this study were familiar with the common side effects associated with the use of contraceptives, as more than three-quarters of them indicated irregular menstruation as one of these effects. Age and years of practice significantly influenced the level of knowledge exhibited by the participants, as older respondents and those with longer years of practice reported higher knowledge scores. This implies that relevant knowledge acquired increased, based on the duration of practice. Pharmacists also reported a higher knowledge score compared to other categories of community pharmacy staff, and this was expected considering their robust training to offer healthcare services [35].

Insights from this study reveals that community pharmacy staff play a significant role in either facilitating or obstructing adolescents' access to necessary healthcare, particularly in the area of contraception. Collectively, close to half of the participants reported moderate and positive attitudes towards contraceptive use whilst the remaining half of the study cohort had negative attitudes. The participants were against providing contraceptives to unmarried adolescents as the majority of them felt this could promote sexual promiscuity. These findings are similar to those of studies amongst other categories of healthcare professionals in Nigeria , and those undertaken in other settings [36-38,43]. Available evidence suggests that adolescents avoid accessing public health facilities due to these negative attitudes, alongside their fear of stigmatisation [44,45]. Young individuals seeking to prevent unwanted pregnancies often face criticism from pharmacy staff when they are merely trying to access essential healthcare services. A considerable proportion of the participants felt that parental consent should be sought before providing contraceptives to people of young age whilst, in essence, there is no provision for this in Nigerian laws. Currently, Nigeria’s reproductive health policies are ambiguous regarding access to contraceptive by adolescents, particularly in community pharmacies, and consequently creating a gap that needs be addressed through clear guidelines and reforms. For government and policymakers, this provides an opportunity to articulate a robust yet contextual framework to guide healthcare workers in the discharge of their responsibilities as it relates to accessing contraceptives by members of the public. Establishing such frameworks, can enable government address the confusion around adolescent contraceptives’ access and ensure that healthcare providers, particularly in community pharmacies, have the necessary guidelines to serve young populations responsibly.

A strong majority of the participants demonstrated good practice in their outlook for the recommendation of contraceptives to both married and unmarried adults. However, a little less than half of the participants opposed providing adolescents with contraceptives. Whilst this may be attributable to issues relating to morality, the phenomenon is worthy of further study. Abstinence from sex represents the best strategy to prevent unwanted pregnancies amongst young people, however, professionals need to be better trained as regards contraceptive access for populations unable to abstain. This is important especially as policy guidelines regarding the provision of contraceptives to unmarried adolescents seem to remain unclear amongst healthcare professionals [36]. This gap in policy guidance necessitates immediate attention from Nigerian authorities to clarify the role of healthcare providers in providing contraception to adolescents, ensuring that all healthcare workers are aligned with national goals.

The commonly recommended contraceptive method indicated by the study participants was the use of condoms, closely followed by the use of oral contraceptive pills. The least recommended were diaphragms, contraceptive rings, and sterilisation. The recommendation pattern may be attributable to the community pharmacy study setting which inadvertently influenced the choice of contraceptive methods easily dispensed within their premises. Further studies that build on these emergent findings also need to be undertaken in other settings, as well as amongst other healthcare professional groups.

Results from the multivariable linear regression analysis suggest that both age and position of the study participants significantly influenced their knowledge of contraception. This finding aligns with several studies [46-48], which also highlight age as a key factor influencing contraceptive knowledge. Additionally, the bivariate linear regression analysis indicates that as knowledge about contraception increases, attitudes toward contraception become more positive. This finding is consistent with a study by Dehlendorf and colleagues [49] which also emphasized the positive relationship between knowledge and attitudes towards contraception

Limitations of this study derive from the convenience sampling strategy adopted to recruit participants, which may not be representative of the population of community pharmacy staff in the Federal Capital Territory. However, this is mitigated by various strengths of the study, which include validation and a robust pre-testing of the research instrument. Another limitation of this study is the potential for self-reporting bias, where participants may provide socially desirable answers or intentionally misrepresent their views to create a favourable impression. This was minimized by using a validated questionnaire.

**Conclusion**

This study reported poor knowledge and negativity in attitudes towards contraception. Although the overall knowledge of the participants was suboptimal, the participants however demonstrated adequate knowledge in certain aspects. Negative attitudes amongst healthcare providers can prevent people from accessing relevant services in the area of reproductive health. The participants appear to be familiar with the common side effects of contraceptives. Age and years of practice significantly influenced the level of knowledge exhibited by participants. Also, pharmacists reported a higher knowledge score compared to other categories of community pharmacy staff. Furthermore, the majority of the participants demonstrated good practice in their outlook for the recommendation of contraceptives to both married and unmarried adults. This study revealed that a little less than half of the participants opposed providing adolescents with contraceptives. The novel findings that emerged from this study can guide government and policymakers in developing relevant strategies that prevent unintended pregnancies amongst the populace.

Clear contextual policy guidelines can enable access to contraceptives for members of the public and prevent discriminative practice among healthcare professionals. Also, his is critically important to provide training for, and build the capacity of healthcare workers as regards service provision for the public in this area. As well as reducing unintended pregnancies, this intervention will also mitigate consequent health complications and associated socio-economic burden.

As this is the first study amongst community pharmacy staff in this setting, the novel findings that emerged provide an empirical basis for contextual policy and practice reforms which can be led by government and other relevant stakeholders. The development of relevant reproductive health guidelines that are fit for purpose in this context can enable improved access to contraceptives in community pharmacies and similar healthcare settings. Further studies can be undertaken to deepen the emergent findings from this study.

**References**

1. Goyal M, Zhao H, Mollen C. Exploring emergency contraception knowledge, prescription practices, and barriers to prescription for adolescents in the emergency department. Pediatrics. 2009 Mar;123(3):765-770. <https://doi.org/10.1542/peds.2008-0193>
2. Alam K, Snover A, Sultana N, Munir TA, Shah SS. Emergency contraception: knowledge, attitude and practices among doctors of a tertiary care hospital. Journal of Ayub Medical College Abbottabad. 2013 Jun 1;25(1-2):141-144.
3. Santelli J, Rochat R, Hatfield-Timajchy K, Gilbert BC, Curtis K, Cabral R, Hirsch JS, Schieve L, Unintended Pregnancy Working Group. The measurement and meaning of unintended pregnancy. Perspectives on sexual and reproductive health. 2003 Mar 1:94-101
4. Centers for Disease Control (2021). Unintended Pregnancy. Available online: <https://www.cdc.gov/reproductivehealth/contraception/unintendedpregnancy/index.htm>. Accessed July 15, 2022.
5. Shakya S, Shrestha S, Shrestha RK Giri U, Shrestha S. Knowledge, attitude and practice of emergency contraceptive pills among community pharmacy practitioners working in Kathmandu Valley: a cross-sectional study. BMC Health Serv Res 2020 Dec;20:699. <https://doi.org/10.1186/s12913-020-05543-5>
6. Singh S, Sedgh G, Hussain R. Unintended pregnancy: worldwide levels, trends, and outcomes. Studies in family planning. 2010 Dec;41(4):241-250. <https://doi.org/10.1111/j.1728-4465.2010.00250.x>
7. Jonathan Marc Bearak, Anna Popinchalk, Cynthia Beavin, Bela Ganatra, Ann-Beth Moller, Özge Tunçalp, Leontine Alkema - Country-specific estimates of unintended pregnancy and abortion incidence: a global comparative analysis of levels in 2015–2019: BMJ Global Health 2022;7:e007151
8. Hanson SJ, Burke AE. "Fertility control: contraception, sterilization, and abortion". In Hurt KJ, Guile MW, Bienstock JL, Fox HE, Wallach EE (eds.). The Johns Hopkins manual of gynecology and obstetrics (4th ed.). Philadelphia: Wolters Kluwer Health/Lippincott Williams & Wilkins. 2010; pp. 382–395.
9. World Health Organisation. Family planning: A global handbook for providers: Evidence-based guidance developed through worldwide collaboration. Geneva. 2011. Available online: <https://fphandbook.org/sites/default/files/hb_english_2012.pdf> (Accessed July 15, 2022)
10. Black AY, Fleming NA, Rome ES. Pregnancy in adolescents. Adolescent medicine: State of the art reviews. 2012 Apr;23(1):123-138
11. Rowan SP, Someshwar J, Murray P. Contraception for primary care providers. Adolescent medicine: state of the art reviews. 2012 Apr 1;23(1):95-110.
12. Cleland J, Conde-Agudelo A, Peterson H, Ross J, Tsui A. "Contraception and health". Lancet. 2012; 380 (9837): 149–156. <https://doi.org/10.1016/S0140-6736(12)60609-6>
13. Oyo-Ita A, Afolabi B, Adedokun B, et al. 'Our culture prohibits some things': qualitative inquiry into sociocultural challenges to scale-up of injectable contraceptives in Nigeria. BMJ Open. 2020;10(7):e035311. Available from: [https://bmjopen.bmj.com/content/10/7/e035311](https://bmjopen.bmj.com/content/10/7/e035311)
14. Sinai I, Nyenwa T, Oguntunde O. Unmet need for family planning and barriers to contraceptive use in northern Nigeria. J Fam Plann Reprod Health Care. 2019;45(3):167-174. Available from: <https://www.tandfonline.com/doi/full/10.1080/13691058.2019.1672894>
15. Udechukwu F, Ezumah N, Ngwu A, Amujiri O, Okafor O. Family planning policy and gender in Nigeria: A thematic analysis of the literature. Ikenga Int J Inst Afr Stud. 2022;23(2):1-12. Available from: <https://www.ikengajournal.com.ng/admin/img/paper/23_2-10.pdf>
16. Fapohunda, B. M., & Orobaton, N. (2013). "Barriers to contraceptive use in Nigeria: A qualitative study." African Journal of Reproductive Health, 17(1), 58-66. This study highlights the role of healthcare providers in creating barriers to contraceptive access through stigmatization and lack of proper information.
17. Akinyemi, J. O., & Adebayo, A. M. (2021). "Healthcare providers’ attitudes and contraceptive use among women in Nigeria: Implications for policy." BMC Health Services Research, 21(1), 1-10. This research emphasizes how healthcare providers' attitudes can impact women's access to contraceptives.
18. Ogunbajo, A., & Oduola, A. (2020). "Barriers to contraceptive use among women in Nigeria: A systematic review." Journal of Public Health in Africa, 11(2), 123-130. This article discusses various barriers to contraceptive access, including healthcare provider biases and inadequate training.
19. Corroon M, Kebede E, Spektor G, Speizer I. Key Role of Drug Shops and Pharmacies for Family Planning in Urban Nigeria and Kenya. Glob Health Sci Pract. 2016 Dec 28;4(4):594-609. doi: 10.9745/GHSP-D-16-00197. PMID: 28031299; PMCID: PMC5199177.
20. Hobbs MK, Taft AJ, Amir LH, Stewart K, Shelley JM, Smith AM, Chapman CB, Hussainy SY. Pharmacy access to the emergency contraceptive pill: a national survey of a random sample of Australian women. Contraception. 2011;83(2):151–158. <https://doi.org/10.1016/j.contraception.2010.06.003>
21. Rafie S, Stone RH, Wilkinson TA, Borgelt LM, El-Ibiary SY, Ragland D. Role of the community pharmacist in emergency contraception counseling and delivery in the United States: current trends and future prospects. Integr Pharm Res Pract. 2017;6:99-108. <https://doi.org/10.2147/IPRP.S99541>
22. Latthe M, Latthe P, Charlton R. Quality of information on emergency contraception on the Internet. Br J Fam Plann. 2000;26(1):39–43.
23. Omotoso O, Ajuwon AJ. Emergency contraceptive pill knowledge, attitudes and dispensing practices of pharmacists in Ibadan and Lagos metropolis, Nigeria. Sierra Leone J Biomed Res. 2010;2(2):135–141.
24. Kasso T, Alegbeleye JO. Knowledge, Attitude and Practice of Contraceptive Use among Women of Reproductive Age in Port Harcourt, Nigeria. Advances in Reproductive Sciences 2023; 11 (4): 93-105. <https://doi:10.4236/arsci.2023.114009>
25. Utoo BT, Mutihir TJ, Utoo PM. Knowledge, attitude and practice of family planning methods among women attending antenatal clinic in Jos, North-central Nigeria. Niger J Med 2010; 19(2):214-8. https://doi:10.4314/njm.v19i2.56524. PMID: 20642092
26. Idowu A, Fehintola FO, Popoola FG. Knowledge, attitude and practice of contraception by female junior secondary school students in an urban community of Oyo-state, South west, Nigeria. Int J Reprod Contracept Obstet Gynecol 2017; 6(11). <https://doi.org/10.18203/2320-1770.ijrcog20174983>
27. Anderson C, Blenkinsopp A. Community pharmacy supply of emergency hormonal contraception: a structured literature review of international evidence. Human Reproduction. 2006 Jan 1;21(1):272-284. <https://doi.org/10.1093/humrep/dei287>
28. Hussainy SY, Stewart K, Chapman CB, Taft AJ, Amir LH, Hobbs MK, Shelley JM, Smith AM. Provision of the emergency contraceptive pill without prescription: attitudes and practices of pharmacists in Australia. Contraception. 2011 Feb 1;83(2):159-166. <https://doi.org/10.1016/j.contraception.2010.07.001>
29. Thaci J, Foster AM. Emergency contraception in Albania: a multimethods qualitative study of awareness, knowledge, attitudes and practices. Contraception. 2018 Aug 1;98(2):110-114. <https://doi.org/10.1016/j.contraception.2018.03.033>
30. Barakat M, Al-Qudah RA, Akour A, Al-Qudah N, Dallal Bashi YH. Unforeseen uses of oral contraceptive pills: Exploratory study in Jordanian community pharmacies. PloS one. 2020 Dec;15(12):e0244373. <https://doi.org/10.1371/journal.pone.0244373>
31. Ekpenyong A, Udoh A, Kpokiri E, Bates I. An analysis of pharmacy workforce capacity in Nigeria. Journal of pharmaceutical policy and practice. 2018 Dec;11(1):1-9.
32. Centers for Disease Control (2022). EPI Info: Stat Calc. Available online: <https://www.cdc.gov/epiinfo/user-guide/statcalc/statcalcintro.html> Accessed February 13, 2024
33. Ahmad H, Halim H. Determining sample size for research activities. Selangor Business Review. 2017 Dec;2(1):20-34.
34. Seid, M. A., & Hussen, M. S. Knowledge and attitude towards antimicrobial resistance among final year undergraduate paramedical students at University of Gondar, Ethiopia. BMC infectious diseases, 2018;18(1), 312. https://doi.org/10.1186/s12879-018-3199-1
35. Azmi NL, Azman NF, Romainor NN, Abdul MH, Rahman MS. Knowledge, Awareness and Perception about Contraception among Pharmacy Staffs: A Single Centre Experience. Pharmacy Research Reports. 2020; 3(1):10-17
36. Ketting, E. & Esin, A. (2010) 'Integrating sexual and reproductive health in primary health care in Europe: position paper of the European Forum for Primary Care', *International Exchange*, 18(4).
37. Hassell K, Rogers A, Noyce P. Community pharmacy as a primary health and self‐care resource: a framework for understanding pharmacy utilization. Health & social care in the community. 2008;8(1):40-49. <https://doi.org/10.1046/j.1365-2524.2000.00222.x>
38. Tonna AP, Weidmann AE, Sneddon J, Stewart D. Views and experiences of community pharmacy team members on antimicrobial stewardship activities in Scotland: a qualitative study. International journal of clinical pharmacy. 2020;42:1261–1269. <https://doi.org/10.1007/s11096-020-01042-z>
39. Federal Ministry of Health (2020) Nigeria’s National Family Planning Blueprint (2020–2024). Abuja: Federal Ministry of Health. Available at: https://policyvault.africa/policy/nigeria-family-planning-blueprint-2020-2024/ (Accessed: 22^nd^ December, 2024)
40. Mngadi PT, Faxelid E, Zwane IT, Höjer B, RansjoArvidson AB. Health providers’ perceptions of adolescent sexual and reproductive health care in Swaziland. Int Nurs Rev 2008; 55: 148–155. <https://doi.org/10.1111/j.1466-7657.2007.00625.x>
41. Mmari KN, Magnani RJ. Does making clinic-based reproductive health services more youth-friendly increase service use by adolescents? Evidence from Lusaka, Zambia. J Adolesc Health 2003; 33: 259-270. <https://doi.org/10.1016/S1054-139X(03)00062-4>
42. Lindberg C, Lewis-Spruill C, Crownover R. Barriers to sexual and reproductive health care: urban male adolescents speak out. Issues Compr Pediatr Nurs 2006; 29: 73-88. <https://doi.org/10.1080/01460860600677577>
43. Abubakar, I. B., and Abubakar, H. B. (2024). Nigerian women’s modern contraceptive use: evidence from NDHS 2018. Reproduction and Fertility 5, 2, e230063, available from: < https://doi.org/10.1530/RAF-23-0063> [Accessed 20 December 2024]
44. Fadeyibi, O., Alade, M., Adebayo, S., Erinfolami, T., Mustapha, F., & Yaradua, S. (2022). Household Structure and Contraceptive Use in Nigeria. Frontiers in Global Women's Health, 3, 821178. <https://doi.org/10.3389/fgwh.2022.821178>
45. Dehlendorf C, Levy K, Ruskin R, Steinauer J. Health care providers' knowledge about contraceptive evidence: a barrier to quality family planning care? Contraception. 2010 Apr;81(4):292-8. doi: 10.1016/j.contraception.2009.11.006. Epub 2009 Dec 11. PMID: 20227544; PMCID: PMC2892417.
